# High contrast cartilaginous endplate imaging in spine using three dimensional dual-inversion recovery prepared ultrashort echo time (3D DIR-UTE) sequence

**DOI:** 10.1007/s00256-023-04503-4

**Published:** 2023-11-08

**Authors:** Jiyo S. Athertya, James Lo, Xiaojun Chen, Soo Hyun Shin, Bhavsimran Singh Malhi, Saeed Jerban, Yang Ji, Sam Sedaghat, Hiroshi Yoshioka, Jiang Du, Monica Guma, Eric Y. Chang, Yajun Ma

**Affiliations:** 1 Department of Radiology, University of California San Diego, San Diego, CA USA; 2 Department of Bioengineering, University of California San Diego, San Diego, CA USA; 3 Department of Radiological Sciences, University of California Irvine, Irvine, CA USA; 4 Radiology Service, Veterans Affairs San Diego Healthcare System, San Diego, CA USA; 5 Department of Medicine, University of California San Diego, San Diego, CA USA; 6 Medicine Service, Veterans Affairs San Diego Healthcare System, San Diego, CA USA

**Keywords:** Cartilaginous endplate, High contrast, Dual adiabatic inversion recovery, UTE, Low back pain

## Abstract

**Purpose:**

To investigate the feasibility and application of a novel imaging technique, a three-dimensional dual adiabatic inversion recovery prepared ultrashort echo time (3D DIR-UTE) sequence, for high contrast assessment of cartilaginous endplate (CEP) imaging with head-to-head comparisons between other UTE imaging techniques.

**Method:**

The DIR-UTE sequence employs two narrow-band adiabatic full passage (AFP) pulses to suppress signals from long T_2_ water (e.g., nucleus pulposus (NP)) and bone marrow fat (BMF) independently, followed by multispoke UTE acquisition to detect signals from the CEP with short T_2_ relaxation times. The DIR-UTE sequence, in addition to three other UTE sequences namely, an IR-prepared and fat-saturated UTE (IR-FS-UTE), a T_1_-weighted and fat-saturated UTE sequence (T_1w_-FS-UTE), and a fat-saturated UTE (FS-UTE) was used for MR imaging on a 3 T scanner to image six asymptomatic volunteers, six patients with low back pain, as well as a human cadaveric specimen. The contrast-to-noise ratio of the CEP relative to the adjacent structures—specifically the NP and BMF—was then compared from the acquired images across the different UTE sequences.

**Results:**

For asymptomatic volunteers, the DIR-UTE sequence showed significantly higher contrast-to-noise ratio values between the CEP and BMF (CNR_CEP-BMF_) (19.9 ± 3.0) and between the CEP and NP (CNR_CEP-NP_) (23.1 ± 1.7) compared to IR-FS-UTE (CNR_CEP-BMF_: 17.3 ± 1.2 and CNR_CEP-NP_: 19.1 ± 1.8), T_1w_-FS-UTE (CNR_CEP-BMF_: 9.0 ± 2.7 and CNR_CEP-NP_: 10.4 ± 3.5), and FS-UTE (CNR_CEP-BMF_: 7.7 ± 2.2 and CNR_CEP-NP_: 5.8 ± 2.4) for asymptomatic volunteers (all *P*-values < 0.001). For the spine sample and patients with low back pain, the DIR-UTE technique detected abnormalities such as irregularities and focal defects in the CEP regions.

**Conclusion:**

The 3D DIR-UTE sequence is able to provide high-contrast volumetric CEP imaging for human spines on a clinical 3 T scanner.

**Supplementary Information:**

The online version contains supplementary material available at 10.1007/s00256-023-04503-4.

## Introduction

Low back pain (LBP) is one of the major causes of disability, affecting approximately 25% of adults in the United States [1, 2]. The need for accurate diagnoses as well as focused preventive and therapeutic strategies in this condition have become a major public health priority [3]. Intervertebral disc (IVD) degeneration has been recognized as one of the main causes of chronic low back pain [4]. Many of these cases have been associated with abnormality of the cartilaginous endplate (CEP) [5].

Vertebral endplate structure lies at the cranial and caudal interface of the intervertebral disc on one side and vertebral body, which consists of an osseous component and a hyaline cartilage component (known as the CEP), on the other [6]. The CEP comprised of a hydrated proteoglycan matrix, fortified by an interlaced framework of collagen fibrils, which directly attaches to the intervertebral disc via the lamellae within the inner (medial) annulus fibrosus. No direct connection is found between CEP and the osseous structure of the vertebral bodies [7].

The CEP acts as the nutrient transport bridge from blood vessels to the disc cells, which is of critical importance to maintaining disc health [8, 9]. Degeneration, mineralization, and dehydration of the CEP due to age, injury, and degradation reduce the CEP’s permeability, leading to a diminished capacity for nutrient transport. Thus, the health of the CEP could be highly related to early IVD degeneration and associated LBP.. Moreover, the CEP is also subjected to mechanical loads which are distributed onto adjacent vertebrae as a means to prevent the disc nucleus from bulging under pressure [10]. Damage to this region might present as a painful pathology [11]. Thus, evaluation of the CEP region may be critical for the assessment of LBP and diagnosis of early IVD degeneration.

Magnetic resonance imaging (MRI) is a powerful tool for the diagnosis of IVD degeneration. The most commonly used techniques are the T_1_- and T_2_-weighted sequences [12]. However, because the CEP has a relatively short transverse relaxation time (T_2_: ~ 18 ms, T_2_*: ~ 15 ms)) [13, 14], these routine clinical sequences cannot detect sufficient CEP signals for direct imaging or quantification, thus limiting the effectiveness of MRI for early evaluation of IVD degeneration.

Ultrashort echo time (UTE) sequences with echo times shorter than 100 µs are able to detect many short T_2_ tissues, such as the CEP, tendons, and bone, positioning this type of sequence as a promising approach to image and evaluate those tissues [15, 16]. Recently, several UTE techniques have been developed for long T_2_ suppression and selective imaging of short T_2_ tissues, such as dual-echo UTE with subtraction [17], T_1_-weighted fat-saturated UTE (T_1w_-FS-UTE) [18], inversion recovery-prepared and fat-saturated UTE (IR-FS-UTE) [19], and dual inversion recovery-prepared UTE (DIR-UTE) [20] sequences. The dual-echo UTE is a fast technique but suffers from low contrast-to-noise ratio (CNR) between the CEP and bone marrow fat (BMF) due to the relatively fast T_2_* decay of BMF [21]. The fast T_1w_-FS-UTE sequence has been applied for high-contrast imaging of the osteochondral junction (OCJ) with better contrast between the CEP and BMF than the dual-echo subtraction method [18]. Moreover, the UTE sequences which incorporate adiabatic inversion, such as IR-FS-UTE, are very efficient in generating high contrast between short and long T_2_ tissues [19, 20, 22–24] and have been used for high contrast imaging of the OCJ [25]. Such techniques can aid in imaging the CEP with high resolution [19]. In CEP imaging, the IR-FS-UTE sequence can produce a very high contrast between the CEP and nucleus pulposus (NP), but the contrast between the CEP and BMF is limited because of the chemical fat saturation (FatSat) module that prohibits efficient fat suppression if more acquisition spokes are used in a repetition time (TR). This inefficient fat suppression can be overcome by replacing the FatSat module with another adiabatic inversion pulse that is centered on the fat frequency, i.e., the DIR-UTE technique [20]. The DIR-UTE sequence has been shown to generate a very high image contrast for the OCJ region with efficient suppression of signals from both the long T_2_ cartilage and marrow fat [20].

In this study, we aim to image the CEP instead of the bony endplate. Bony endplate and subchondral bone structures are dark in the proposed DIR-UTE images because cortical bone typically has a much lower proton density than cartilage tissues including the CEP [26, 27]. Given the advantages of DIR-UTE sequence in imaging of short T_2_ tissues, we proposed to further optimize it for high contrast imaging of CEP in the human lumbar spine, and compare its performance with other established UTE techniques, namely IR-FS-UTE, T_1w_-FS-UTE, and FS-UTE.

## Methods

### MR acquisition and pulse sequence

This study was approved by the institutional review board. All sequences were implemented on a 3 T MR750 scanner (GE Healthcare Technologies, Milwaukee, Wisconsin) with a four-channel phased array spine coil utilized for signal reception.

**Fig. 1:**
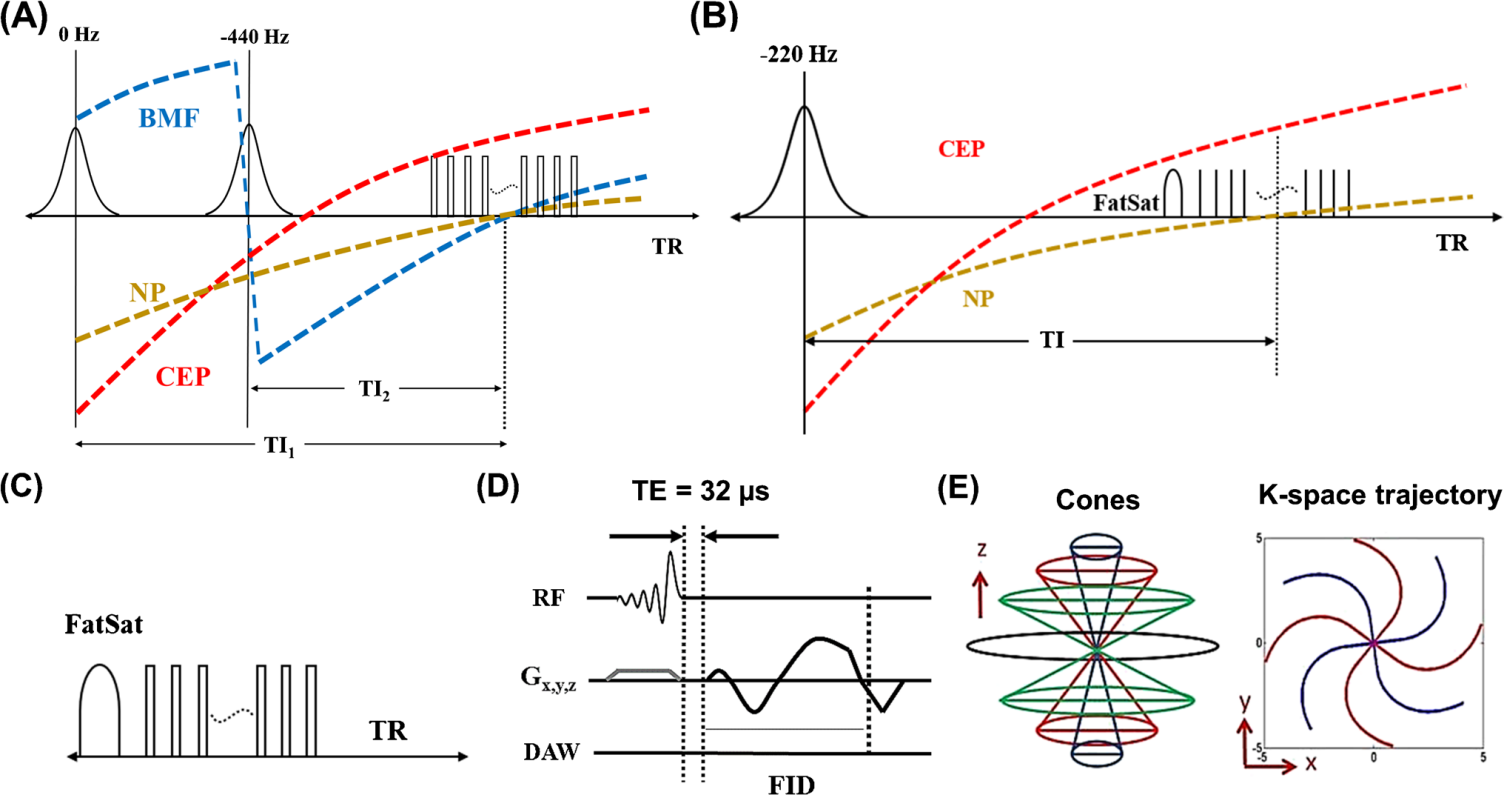
Sequence diagram for four different UTE techniques, including DIR-UTE (**A**) IR-FS-UTE (**B**) T_1w_-FS-UTE (**C**), and FS-UTE (**C**). The DIR-UTE sequence utilizes two AFP pulses to invert long T_2_ water (e.g., NP) and BMF with center frequencies of 0 and -440 Hz, respectively (**A**). The IR-FS-UTE sequence employs an AFP pulse for inverting long NP while the FatSat module is utilized to improve CEP contrast against BMF (**B**). The FatSat module is applied for fat suppression in both T_1w_-FS-UTE and FS-UTE sequences (**C**). The multispoke acquisition strategy is employed in all UTE sequences to reduce the total scan time. A slab selective half pulse is utilized for signal excitation in each spoke (**D**). The 3D Cones trajectory enables efficient k-space coverage for all UTE scans (**E**)

Figure 1 shows the sequence diagrams for the four different UTE techniques used in this study, including DIR-UTE, IR-FS-UTE, T_1w_-FS-UTE, and FS-UTE sequences. The DIR-UTE sequence utilizes two adiabatic full passage (AFP) pulses to invert long T_2_ water (e.g., NP) and BMF with center frequencies of 0 and -440 Hz, respectively [20]. The IR-FS-UTE sequence employs an AFP pulse for inverting long T_2_ NP while the FatSat module is utilized to improve CEP contrast against BMF [19]. The T_1w_-FS-UTE shares the same sequence diagram with the FS-UTE but with much higher flip angles (thus stronger T_1_ weighting) [18]. The FatSat module is applied for fat suppression in both T_1w_-FS-UTE and FS-UTE sequences. The multispoke acquisition strategy is employed in all UTE sequences to reduce the total scan time. For signal excitation in each spoke, a slab selective half pulse (Shinnar-Le Roux design, duration 1132 μs, and bandwidth 16 kHz) with variable-rate selective excitation (VERSE) design [28] is utilized. The 3D Cones trajectory enables efficient k-space coverage for all UTE scans.

In the DIR-UTE sequence, the longitudinal magnetizations of NP and BMF are efficiently inverted by two narrow-band AFP (e.g., 500 Hz) pulses. With a proper selection of inversion time (TI) for each inversion, signal nulling for both NP and BMF could be reached at the time of data acquisition simultaneously [22]. On the other hand, the inverted CEP signal recoveries quickly because of the very short T_1_ property of CEP (~ 400 ms) [19]. When the data acquisition commences around the signal nulling point of the NP and BMF, the CEP presents with high signal and is therefore highlighted.

### In vivo human spine imaging

Twelve volunteers (six asymptomatic subjects and six patients with LBP) were recruited for lumbar spine imaging using both clinical (i.e., T_1w_- and T_2w_-FSE) and UTE (i.e., DIR-UTE, IR-FS-UTE, T_1w_-FS-UTE, and FS-UTE) sequences. The age of asymptomatic subjects ranged between 23 and 38 years old, while that of the patient population ranged between 37 and 60 years old. Informed consent was obtained from all participants per institutional review board requirements.

To determine the optimal TIs for high contrast CEP imaging, DIR-UTE scans were performed on a single asymptomatic volunteer with a series of different TI_1_s and TI_2_s (TIs can be seen in Supplemental Information Table 1). The other major parameters for each scan were TR/echo time (TE) = 1500/0.032 ms, flip angle (FA) = 10^°^, number of spokes per TR(N_sp_) = 27, field-of-view (FOV) = 28 × 28 × 4.8 cm^3^, matrix = 220 × 220 × 12, voxel size = 1.273 × 1.273 × 4 mm^3^, bandwidth = 125 kHz, and scan time = 6 min.

The remaining asymptomatic volunteers and symptomatic patients were scanned with the resultant optimized protocol: (i) DIR-UTE: TR/TI_1_/TI_2_ = 1500/610/150 ms, TE = 0.032 ms, FA = 10^°^, N_sp_ = 27, FOV = 28 × 28 × 4.32 cm^3^, matrix = 320 × 320 × 12, voxel size = 0.875 × 0.875 × 3.6 mm^3^, bandwidth = 125 kHz, and scan time = 10 min; (ii) IR-FS-UTE [19]: TR/TI = 1200/600 ms, TE = 0.032 ms, FA = 10^°^, N_sp_ = 21, FOV = 28 × 28 × 4.32 cm^3^, matrix = 320 × 320 × 12, voxel size = 0.875 × 0.875 × 3.6 mm^3^, bandwidth = 125 kHz, and scan time = 10 min; (iii) T_1w_-FS-UTE: TR = 120 ms, TE = 0.032 ms, FA = 15^°^, FOV = 28 × 28 × 4.32 cm^3^, matrix = 320 × 320 × 12, voxel size = 0.875 × 0.875 × 3.6 mm^3^, bandwidth = 125 kHz, and scan time = 2 min 20 s; (iv) FS-UTE: TR = 120 ms, TE = 0.032 ms, FA = 6^°^, FOV = 28 × 28 × 4.32 cm^3^, matrix = 320 × 320 × 12, voxel size = 0.875 × 0.875 × 3.6 mm^3^, bandwidth = 125 kHz, and scan time = 2 min 20 s. Clinical T_1w_- and T_2w_-FSE sequences were scanned for comparison. In addition, for the patient scan, a proton density weighted-UTE (PDw-UTE) sequence with an isotropic resolution (1.2 mm^3^) was also employed for high contrast cortical bone imaging [29]. The sequence parameters used in the other UTE sequences were optimized in previous studies.

### Ex vivo human spine imaging

A cadaveric spine sample section (T11-T12) from an 87-year-old female donor was scanned using the DIR-UTE sequence with the following parameters: TR/TI_1_/TI_2_ = 1500/610/150 ms, TE = 0.032 ms, FA = 10^°^, N_sp_ = 27, FOV = 12 × 12 × 4 cm^3^, matrix = 320 × 320 × 20, voxel size = 0.375 × 0.375 × 2 mm^3^, bandwidth = 83.3 kHz, scan time = 16 min. An eight channel-knee coil was used for radiofrequency (RF) transmission and signal reception. Clinical T_1w_- and T_2w_-FSE sequences were scanned for comparison.

### Data processing

**Fig. 2:**
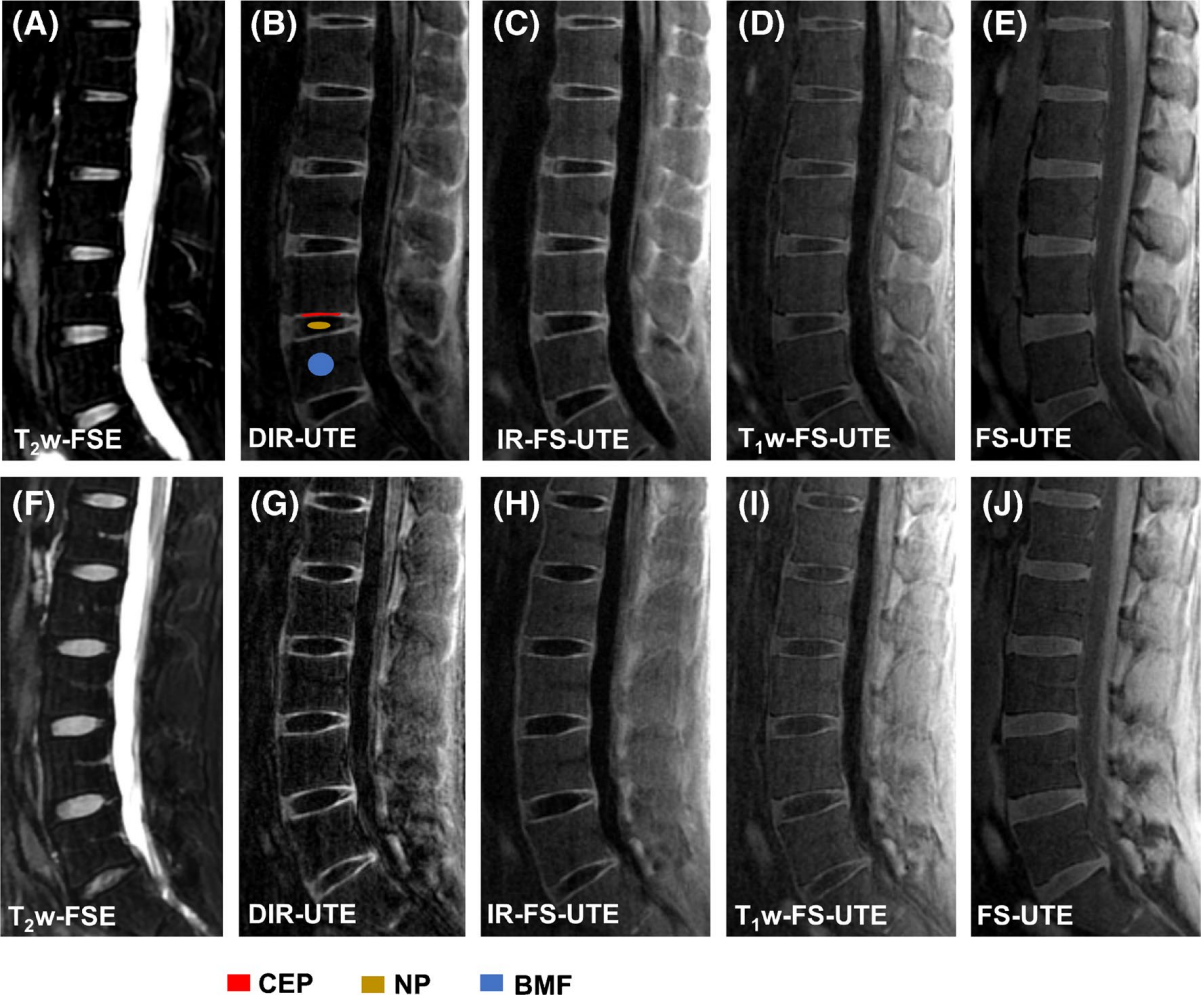
Lumbar spine imaging for two asymptomatic volunteers. Images from a 32-year-old asymptomatic male subject are shown in (**A**—**E**) while (**F**—**J**) correspond to images obtained from a 38-year-old asymptomatic male volunteer. The clinical fat suppressed T_2w_-FSE sequence shown in (**A** and **F**) fails to capture CEP signals owing to its long TE relative to the CEP’s short T_2_ relaxation. The DIR-UTE (**B** and **G**) provides the best contrast in comparison to other UTE imaging techniques, including IR-FS-UTE (**C** and **H**), T_1w_-FS-UTE (**D** and** I**), and FS-UTE (**E** and **J**), between CEP and BMF and between CEP and NP. Panel **B** shows representative ROIs of CEP, NP and BMF to calculate CEP CNRs

To compare the CEP contrast between the four different UTE techniques on asymptomatic volunteers, the CNRs between the CEP and NP (CNR_CEP-NP_) and between the CEP and BMF (CNR_CEP-BMF_) were calculated as the mean differences in signals between these tissues divided by the background noise. Representative ROIs of CEP, NP and BMF can be seen in Fig. 2. The noise was estimated as the standard deviation of signals measured from a region of interest (ROI) (~ 6 × 6 cm^2^) in an artifact-free background region. Descriptive statistics were performed while mean and standard deviation of the CNR_CEP-NP_ and CNR_CEP-BMF_ were measured to evaluate contrast between CEP and NP and between CEP and BMF for all the UTE sequences. Paired t-test using SPSS software (IBM, Armonk, NY, USA) version 28.0 was utilized to compare CNR values of CEP imaging between all the techniques. P-values lower than 0.05 are considered as significant.

## Results

Supplemental Information Fig. 1 shows the DIR-UTE images acquired from a 32-year-old asymptomatic male volunteer at various TI combinations. To optimize the image contrast between CEP and NP, TI_1_ varies from 580 to 640 ms while TI_2_ is fixed at 150 ms. Similarly, to optimize the contrast between CEP and BMF, TI_2_ varies from 125 to 175 ms while TI_1_ is fixed at 610 ms. As can be seen from the figure, the images resulting from these different TI combinations all show good CEP contrast visually, proving that the DIR-UTE technique can effectively highlight the CEP region over a wide range of TI_1_s and TI_2_s. This demonstrates the robustness of the DIR-UTE technique for high contrast imaging. Supplemental Information Table 1 lists the measured CNR_CEP-NP_ and CNR_CEP-BMF_ values for the images shown in Supplemental Information Fig. 1. When TI_2_ is fixed at 150 ms, the CNR_CEP-NP_ values increase from 17.8 to 19.0, then decrease to 17.9 when TI_1_ increases from 580 to 640 ms. In addition, when TI_1_ is fixed at 610 ms, the CNR_CEP-BMF_ values increase from 17.0 to 17.6, then decrease to 15.9 when TI_2_ increases from 125 to 175 ms. The highest CNR_CEP-NP_ and CNR_CEP-BMF_ values are achieved when TI_1_ = 610 ms and TI_2_ = 150 ms. These optimized TIs were employed for the subsequent DIR-UTE imaging of both asymptomatic volunteers and patients.

Figure 2 shows the representative lumbar spine images from two asymptomatic volunteers. The CEP signal cannot be efficiently captured in the clinical fat suppressed T_2w_-FSE sequence owing to its relatively short T_2_ relaxation time, whereas it is clearly seen on all UTE images. The DIR-UTE, IR-FS-UTE, and T_1w_-FS-UTE sequences all produce higher CEP contrast than the regular FS-UTE sequence because of their higher T_1_-weighting property. The DIR-UTE images show the best CEP contrast for visual comparison.

Table 1 summarizes the measured CNR_CEP-NP_ and CNR_CEP-BMF_ values of all four UTE sequences for the six asymptomatic volunteers. Of the different sequences used, the DIR-UTE presents the highest CEP contrast for CEP vs. NP (23.1 ± 1.7) and CEP vs. BMF (19.9 ± 3.0), followed by IR-FS-UTE, T_1w_-FS-UTE, and FS-UTE. The DIR-UTE showed significantly higher CNRs in both CNR_CEP-NP_ and CNR_CEP-BMF_ compared to IR-FS-UTE (*p* = 0.0005, *p* = 0.0109), T_1w_-FS-UTE (*p* = 0.0017, *p* = 0.0003), and FS-UTE (*p* = 0.0010, *p* = 0.0008) respectively.

**Table 1:** Summary of the mean CNR_CEP-NP_ and CNR_CEP-BMF_ measurements from the DIR-UTE, IR-FS-UTE, T_1w_-FS-UTE, and FS-UTE images for six asymptomatic volunteers

CNR	DIR-UTE	IR-FS-UTE	T_1w_-FS-UTE	FS-UTE
Between CEP and NP	23.1 ± 1.7	19.1 ± 1.8	10.4 ± 3.5	5.8 ± 2.4
Between CEP and BMF	19.9 ± 3.0	17.3 ± 1.2	9.0 ± 2.7	7.7 ± 2.2

*BMF* Bone marrow fat; *NP* Nucleus pulposus

**Fig. 3:**
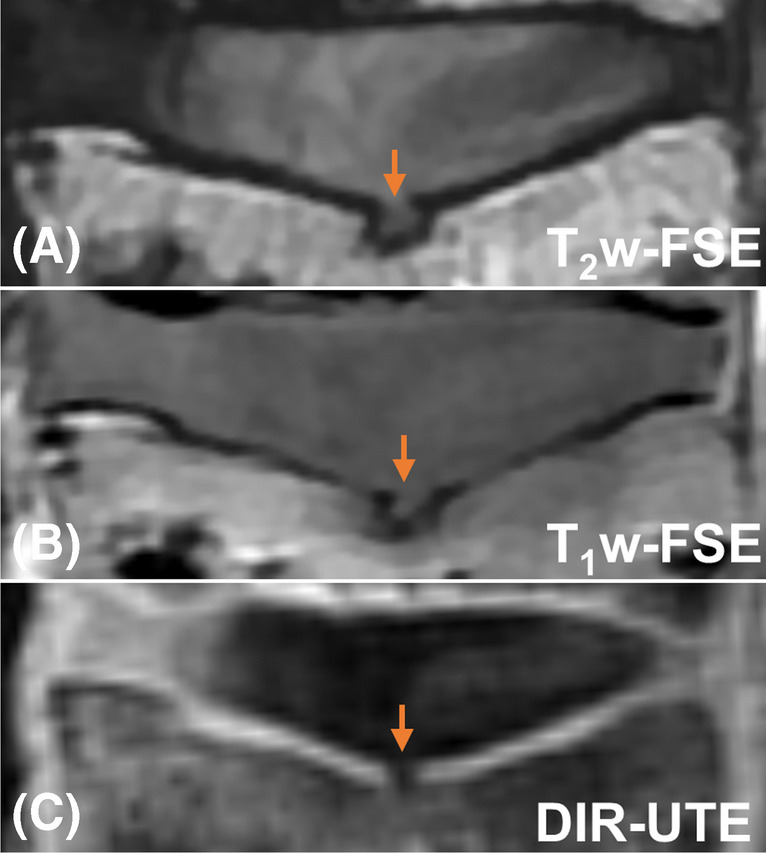
The clinical T_2w_- and T_1w_-FSE (**A** and **B**) as well as DIR-UTE (**C**) imaging for an ex vivo spine sample from an 87-year-old female donor. There is a CEP fracture with herniation of NP through the focal defect as indicated on the DIR-UTE image with an orange arrow

Results from the ex vivo sample study are presented in Fig. 3. As seen from Fig. 3C, signals from long T_2_ water and fat are well suppressed by the DIR technique. The high-intensity band of CEP demonstrates a thickness from 0.6 mm to 1.2 mm. In comparison, the CEP is dark in both clinical T_2w_- and T_1w_-FSE images due to its short T_2_. The lower level of this disc is particularly interesting, with the presence of a compression fracture deformity and central Schmorl’s node at the superior endplate of the lower spine (orange arrows). This focal CEP fracture is clearly evident as a disruption in the high signal band as shown on the DIR-UTE image, but is ambiguous in the clinical T_2w_- and T_1w_- FSE images.

**Fig. 4:**
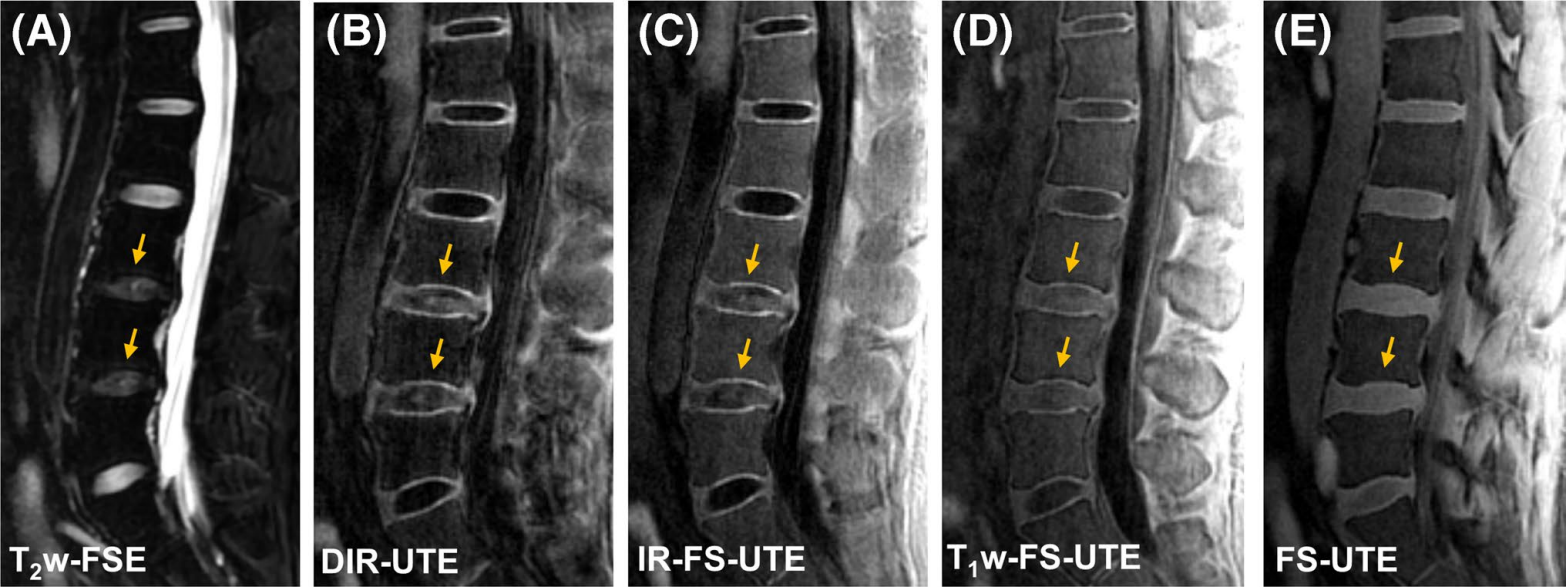
Representative lumbar spine imaging from a 40-year-old male patient with low back pain. While the CEP region is completely invisible in the clinical fat suppressed T_2w_-FSE (**A**) it is well highlighted in the DIR-UTE (**B**) IR-FS-UTE (**C**) and T_1w_-FS-UTE (**D**). Regular FS-UTE (**E**) is able to capture the CEP signal, but has relatively low contrast of the CEP region compared to the other UTE imaging techniques. The NP regions of degenerated discs (indicated by arrows) exhibit stronger signals than those in normal discs for the DIR-UTE, IR-FS-UTE, and T_1w_-FS-UTE, which can be attributed to the shortened T_1_ relaxation due to dehydration in NP

Figure 4 shows representative lumbar spine images from a patient with low back pain. Similar to the case with the asymptomatic subjects, the clinical fat suppressed T_2w_-FSE sequence does not capture signals from the CEP region, while the DIR-UTE, IR-FS-UTE, and T_1w_-FS-UTE images present better CEP contrast than regular FS-UTE. The NP regions of degenerated discs in the DIR-UTE, IR-FS-UTE, and T_1w_-FS-UTE images show relatively higher signals than those in normal discs. This may be because of shortened T_1_ relaxation time in the NP due to disc dehydration [8, 30, 31].

**Fig. 5:**
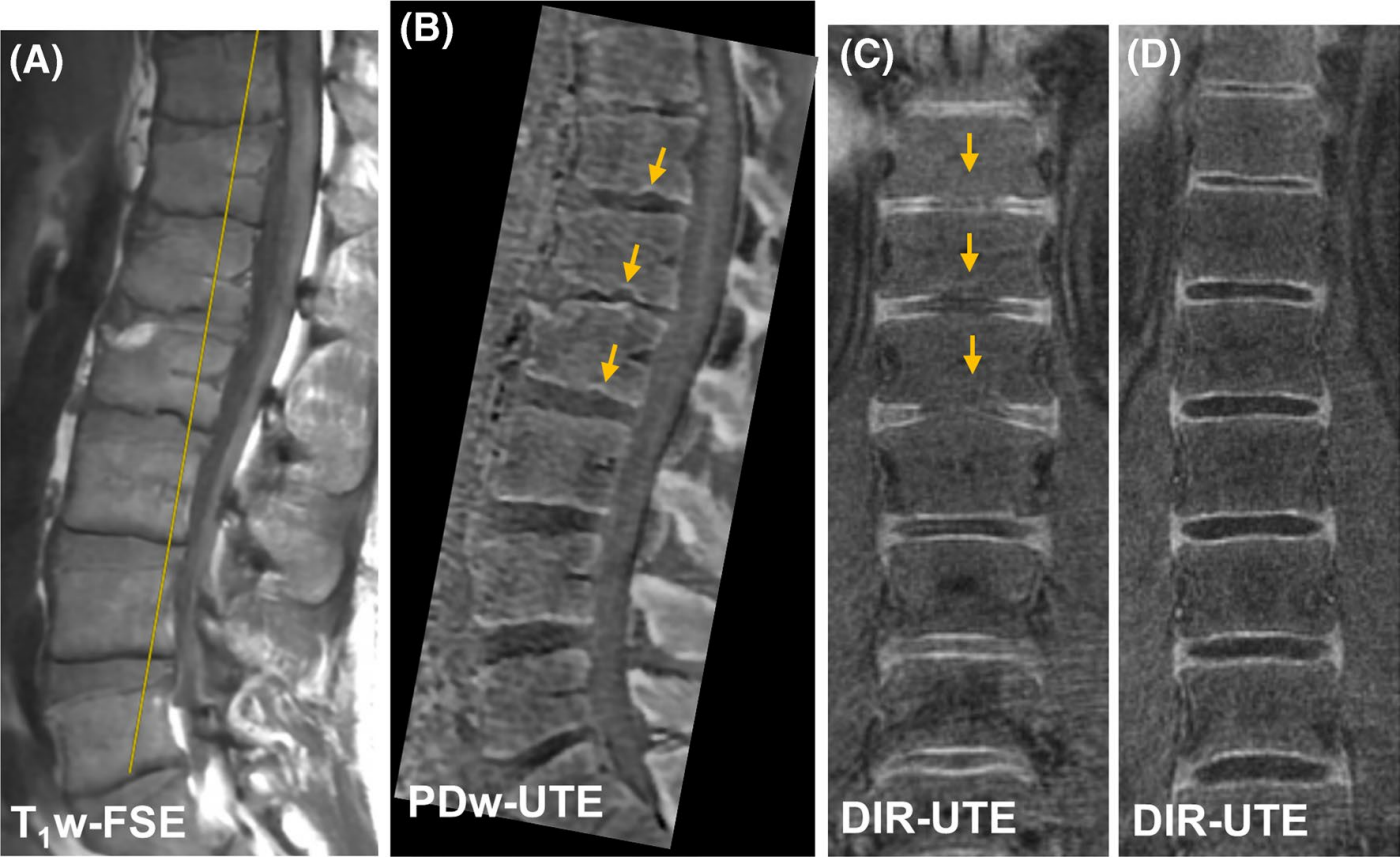
The clinical T_1__w_-FSE (**A**) PDw-UTE (**B**) and DIR-UTE (**C**) images from a 37-year-old male with known PsA. Yellow line in (**A**) on the clinical image indicates the cross-section plane for coronal slice in DIR-UTE image. The CEP irregularities as marked by arrows are depicted in the coronal DIR-UTE image (**C**). The DIR-UTE image obtained from another asymptomatic subject (24-year-old male) (**D**) is used for comparison where the CEP region shows continuous and bright signal

Figure 5 shows UTE imaging of a patient with psoriatic arthritis (PsA). This patient has features of chronic inflammatory arthritis, including a Romanus lesion at the anterosuperior L2 vertebral body with erosion (shown in PDw-UTE bone image) and fatty change (shown in clinical T_1__w_-FSE image). Numerous bony endplate irregularities as marked by arrows are associated with the signal loss in CEP regions seen vividly in the coronal DIR-UTE image (Fig. 5C). In comparison, continuous CEP signals can be seen in the coronal DIR-UTE image from a asymptomatic subject (Fig. 5D).

**Fig. 6:**
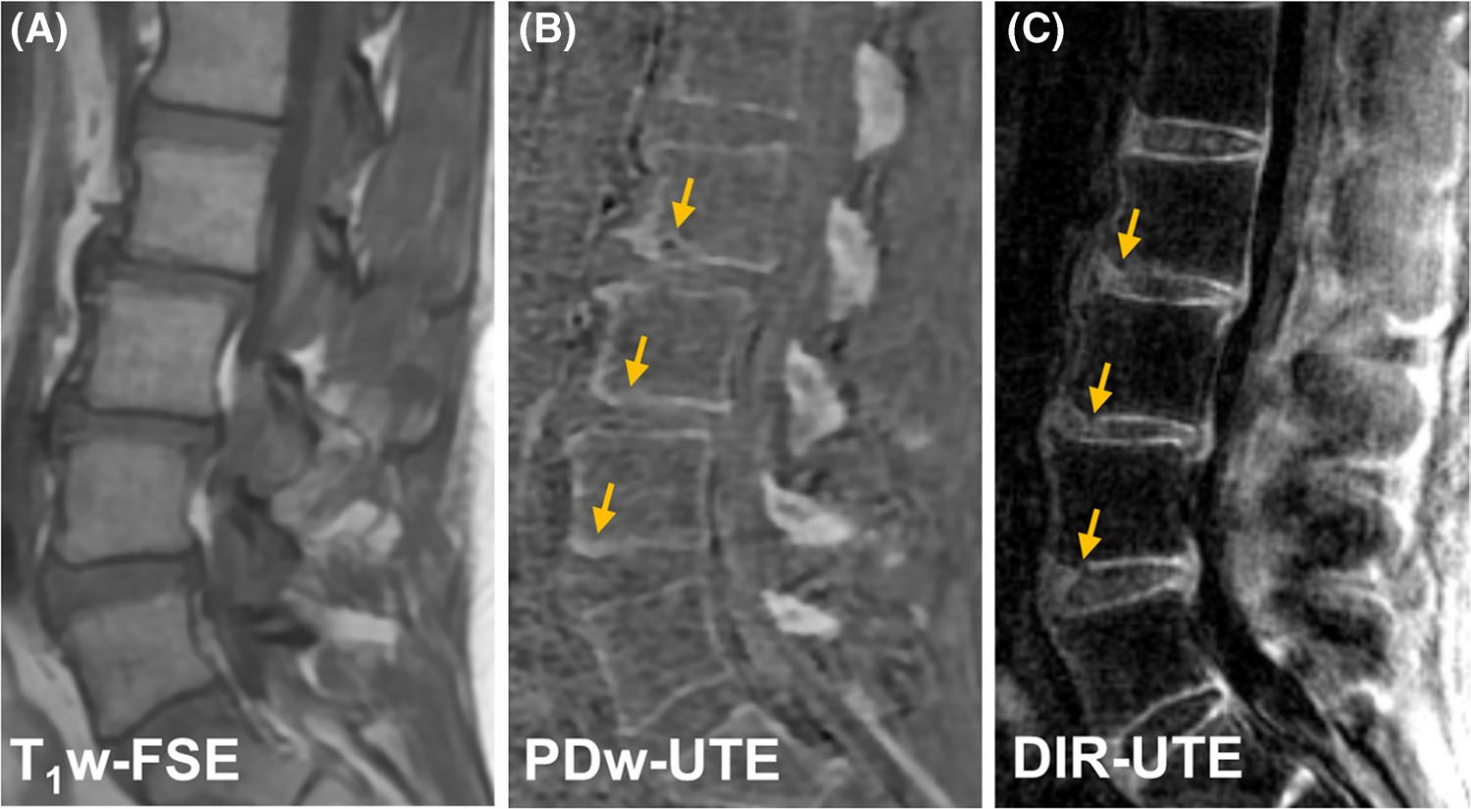
The clinical T_1__w_-FSE (**A**) PDw-UTE (**B**) and DIR-UTE (**C**) images from a 51-year-old male patient with PsA. The CEP irregularities are clearly seen on the inferior endplates at L2, L3, and L4 with the DIR-UTE sequence. These CEP irregularities are associated with bony endplate remodeling shown in the PDw-UTE bone image and were caused by Schmorl’s nodes

Figure 6 presents DIR-UTE imaging of another patient with PsA. The L2-L4 levels show bony endplate remodeling on the PDw-UTE bone image. The CEP irregularities can be clearly seen in the corresponding DIR-UTE image at the inferior endplates of these discs (arrows). In comparison, the clinical sequences do not provide such information on CEP changes.

## Discussion

In this work, we have shown that the DIR-UTE sequence can provide high contrast imaging of the CEP region in both ex vivo and in vivo spine studies. Usage of two narrow-band AFP pulses in the DIR-UTE sequence can efficiently suppress long T_2_ water and fat simultaneously. Moreover, the CEP region can be imaged with high contrast over a wide range of TIs in the DIR-UTE, which indicates the robustness of the proposed technique. In addition, the DIR-UTE sequence highlights the CEP with significant improvements in comparison to IR-FS-UTE, T_1w_-FS-UTE, and FS-UTE sequences. Ex vivo imaging on a spine sample revealed a compression fracture that was clearly discernible by the DIR-UTE sequence. In vivo study on various asymptomatic and symptomatic subjects has confirmed that DIR-UTE sequence can capture CEP signal with high contrast and can detect abnormalities such as CEP irregularities and focal defects.

The proposed 3D DIR-UTE sequence provides better CEP contrast in comparison to previously reported dual-echo subtraction UTE and IR-FS-UTE studies, especially with regard to the contrast between the CEP and BMF [19, 32, 33]. To the best of our knowledge, this is the first in vivo CEP imaging study that uses the 3D DIR-UTE Cones technique; other studies have been focused on the T_2_ * quantification of CEP using multi-echo UTE sequences [14, 34, 35]. Our study has demonstrated that this technique has great potential for morphological evaluation of the CEP and IVD health in vivo. The DIR-UTE technique incorporated with multiple-echo acquisition also allows for quantitative evaluation of CEP degeneration or calcification, which could be a new direction for future investigations.

The CEP region is an important facilitator in both biomechanical and nutritional functions of the spine. Given that the CEP is regularly subjected to significant loads during daily activities in order to stabilize spinal posture [36], end plate disruptions are likely to disrupt the uniformity of disc stress distributions [37]. This in turn accelerates matrix degradation in the CEP thereby causing the impediment of nutrient transport to the cells [38]. This phenomenon can be interpreted as the initial stages of disc degeneration and would be highly significant as a marker observable via noninvasive imaging techniques [5]. Given that conventional MRI sequences fail to capture sufficient signals from the CEP region, devising and developing novel high contrast imaging sequences, such as the DIR-UTE presented in this study, are of acute importance.

The IR preparation is much more efficient in signal suppression for the desired tissues with long T_2_ relaxations than T_1_ weighting (as used in the T_1w_-FS-UTE sequence) is, with regard to short T_2_ imaging. Moreover, since the CEP and BMF both have relatively short T_1_ values, it is difficult to distinguish the regions using the T_1w_-FS-UTE sequence when fat suppression is not complete. In addition, the effectiveness of the FatSat module is limited up to a certain level in the IR-FS-UTE and T_1w_-FS-UTE imaging. When more spokes are utilized to speed up the imaging acquisition, the effectiveness of fat suppression for the FatSat module diminishes, resulting in a trade-off between scan efficiency and fat suppression. This fat suppression inefficiency can be avoided by replacing the FatSat module with another AFP pulse dedicated specifically to fat suppression by centering the frequency on fat.

The DIR-UTE sequence is able to detect and highlight CEP signals, allowing for more accurate morphological evaluation of CEP health in comparison to clinical MRI. For example, calcification and ossification of the CEP cannot be assessed by clinical MRI because these conditions show a similar dark appearance as normal CEP. In comparison, the DIR-UTE may be able to differentiate normal CEP from calcified and ossified CEPs due to the differences in proton density. In addition to morphological assessment of CEP health, the DIR-UTE with multiple-echo acquisition allows quantitative evaluation (i.e., T_2_* measurement) of CEP biochemistry which clinical MRI cannot. Thus the DIR-UTE technique has the potential to provide more useful information than clinical MRI in the assessment of CEP and IVD health.

However, the DIR-UTE imaging could be affected by artifacts brought on by the signal fluctuations among various acquisition spokes if there is a significant increase in the number of spokes employed in one TR. For the current study, the number of spokes (N_sp_ = 27) was found to provide a high CNR without significant artifacts being introduced. A range of 15 to 30 for N_sp_ is recommended in terms of scan time and image quality.

There are several limitations in this study. First, histology was not performed to validate the signal changes of 3D DIR‐UTE imaging. Second, the spatial resolution (e.g., voxel size = 0.875 × 0.875 × 3.6mm^3^) was still a limiting factor for thin CEP imaging considering the signal-to-noise ratio performance of the RF coils and MRI scanners currently available, despite the high CEP contrast in the 3D DIR-UTE imaging. Third, the scan time of 10 min is relatively long in the context of a clinical setting and should be improved with parallel imaging and compressed sensing [39–41]. Fourth, the DIR-UTE sequence is sensitive to background magnetic field inhomogeneity due to narrow bandwidth of the AFP pulses and limited chemical shift of fat at 3T. A high order shimming hardware system could potentially help for uniform signal suppression. Fifth, a relatively small number of subjects were scanned in this study. A future large cohort study will be very useful for exploring the potential of the DIR-UTE technique in clinical use. Sixth, in this study, only the DIR-UTE technique was applied for the patient scans to shorten the total scan time since these patients cannot bear a very long scan due to their moderate low back pain. The comparison of CEP contrast between all the UTE techniques for patient study will be interesting once the scan time of these UTE scans is accelerated.

In conclusion, we showed that 3D DIR-UTE sequence can be used for high contrast CEP imaging both ex vivo and in vivo. The optimized 3D DIR-UTE sequence also showed better CEP contrast than other UTE techniques, including the IR-FS-UTE, T_1w_-FS-UTE, and FS-UTE sequences, suggesting that the DIR-UTE sequence may facilitate better evaluation of the vital CEP region in clinical practice.

### Clinical Relevance

The proposed DIR-UTE sequence could potentially assist in characterizing signal variations in CEP owing to its high CNR. Evaluation of abnormalities in the other human spine tissues, such as facet joints, longitudinal ligaments, and intervertebral foramina, are further potential applications.

### Supplementary Information

Below is the link to the electronic supplementary material.

Supplementary file1 (DOCX 449 KB)

## Data Availability

Data supporting the reported results can be provided by the corresponding author upon courtesy request.

## Abbreviation

LBP: Low back pain
IVD: Intervertebral disc
CEP: Cartilaginous endplate
UTE: Ultrashort echo time
OCJ: Osteochondral junction
T_1w_-FS-UTE: T_1_-weighted fat-saturated UTE
IR-FS-UTE: Inversion recovery-prepared and fat-saturated UTE
DIR-UTE: Dual inversion recovery-prepared UTE
CNR: Contrast-to-noise ratio
BMF: Bone marrow fat
AFP: Adiabatic full passage
TI: Inversion time
VERSE: Variable-rate selective excitation
