# Supplementary material for: High contrast cartilaginous endplate imaging in spine using three dimensional dual-inversion recovery prepared ultrashort echo time (3D DIR-UTE) sequence
Source: Skeletal Radiol. 2023 Nov 8;53(5):881–90. doi: 10.1007/s00256-023-04503-4 (PMC10973042; doi:10.1007/s00256-023-04503-4)
Supplement: Supplementary file 1 — Supplementary file1 (DOCX 449 KB) [file 256_2023_4503_MOESM1_ESM.docx]

**Supplemental Material**

**Supplemental Information Table 1.** Measured CNR_CEP-NP_ and CNR_CEP-BMF_ values with different TIs for optimizing DIR-UTE in high contrast CEP imaging.

| **CEP vs NP** | **TI_2_ = 150 ms** | | |
| --- | --- | --- | --- |
|  | TI_1_ = 580 ms | TI_1_ = 610 ms | TI_1_ = 640 ms |
|  | 17.8±3.2 | 19.0±1.9 | 17.9±0.8 |
| **CEP vs BMF** | **TI_1_ = 610 ms** | | |
|  | TI_2_ = 125 ms | TI_2_ = 150 ms | TI_2_ = 175 ms |
|  | 17.0±2.2 | 17.6±1.6 | 15.9±2.3 |

*BMF: bone marrow fat; NP: nucleus pulposus*


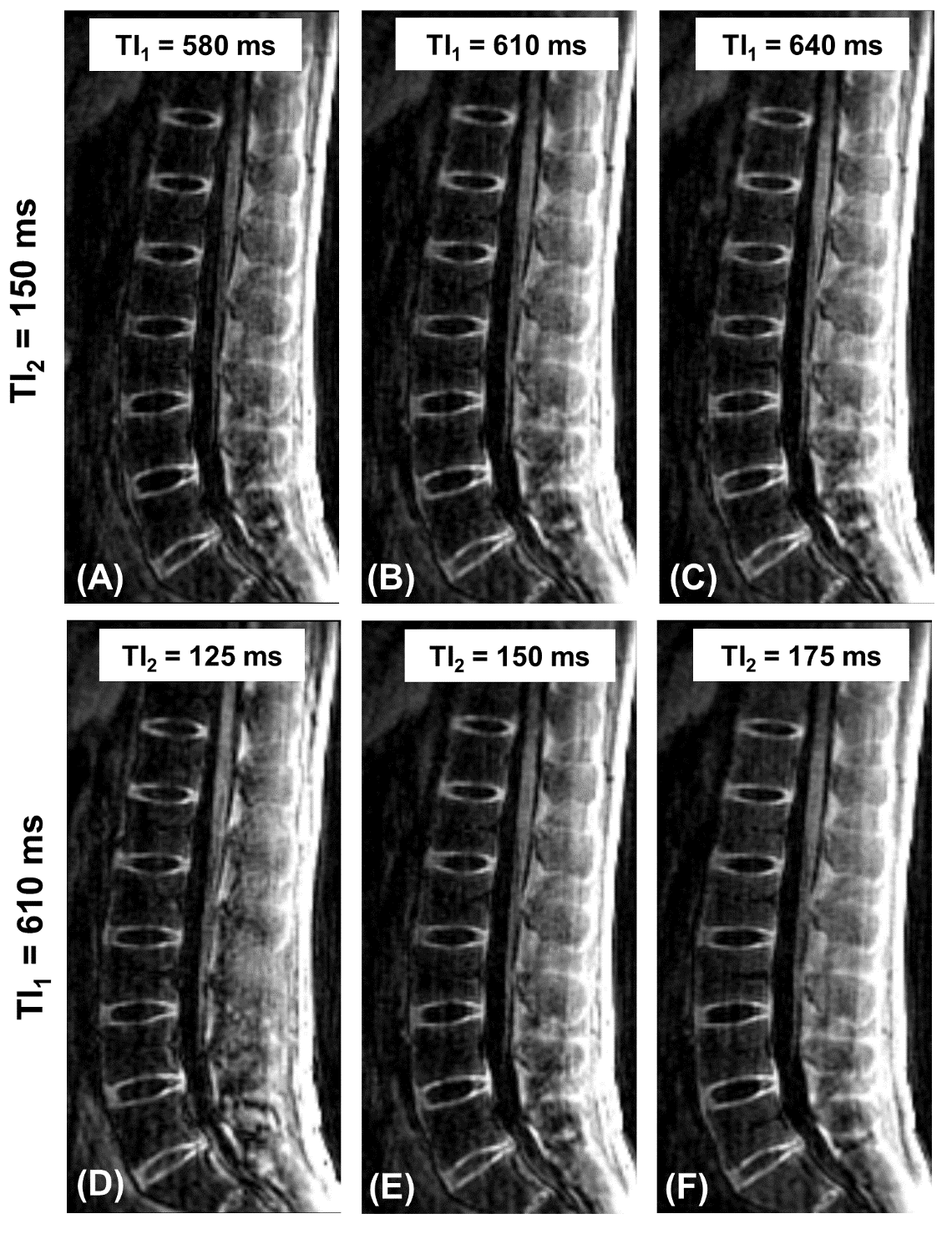


**Supplemental Information Figure 1.** DIR-UTE CEP imaging with different TIs for a 32-year-old asymptomatic male volunteer. (**A**-**C)** compare the contrast between CEP and NP for TI_1_ values of 580, 610, and 640 ms with TI_2_ fixed at 150 ms. (**D**-**F)** compare the contrast between CEP and BMF for TI_2_ values of 125, 150, and 175 ms with TI_1_ fixed at 610 ms.
